# Atlanta’s Potable Waters

**Published:** 1883-07

**Authors:** 


### ATLANTA'S POTABLE WATERS.

I have taken considerable pains to examine some of the most popular wells and the hydrant water of Atlanta. The facts reached are interesting, if not of grave importance to all concerned.

But before going into any topographical particulars, it will be well to present some general information on the subject of potable waters for cities. There are several sources from which this may be derived.

They may come from springs, from deep or shallow wells, from rivers or streams supplied by upland surface water of wild or cultivated lands. Sewage may, also, find access to the latter. Spring and deep well water is naturally both palatable and wholesome.

The water of cisterns and rivers may be sufficiently palatable, but is always suspicious as regards health. Shallow wells and the water of streams to which sewage finds its way are always dangerous.

The people of Atlanta are interested largely, at present, in two of these sources of drinking water; viz: deep wells, and hydrant water derived from a neighboring river.

It is interesting to note, that the older a city grows, the greater becomes a sort of antagonism between the interest of its householders on the one hand and that of its manufacturers and laundries on the other. Househoulders require wholesome water, whether soft or hard, (and both may be equally so), from the present sources, such as springs or deep wells. But this water is usually the hardest, and ill suited to the operations of washing and machinery.

The order in which waters may be classified in regard to softness is, first, rain water; second, upland surface water; third, surface water from cultivated land; fourth, polluted river water; fifth, spring water; sixth, deep well water, and seventh, shallow well water the hardest of all.

The chemical character of a water, however, is largely due to the sort of geologoical formation—the earth and rocks from which it flows. It washes from these in its pathway the mineral ingredients of potash, soda, lime, or magnesia, all, usually in combination with carbonic and sulphuric acids. The salts of the first two give softness to the water, while those of the last make it hard.

The waters of Atlanta, and surrounding country, even those from deep wells and springs are usually soft for the reason already given, and are, therefore, admirably adapted to both domestic, and manufacturing purposes. There are here and there, specimens of water in and around the city which are hard from the presence of magnesia in those places, but these are exceptions.

The city of Atlanta stands upon a foundation of metamorphic granite, consisting mainly of disintegrated feldspar, having little sand, and more or less iron. It is the oxide of iron in this mixture, which gives the soil as seen turned up in the streets, and thrown from the bottom of deep wells and excavations, its characteristic yellow or purplish color.

If this discoloration were absent, together with the small proportion of sand present, the larger part of the city's site would be a vast bed of the purest white porcelain clay, for this disintegrated feldspar, when impalable like flour, and free from metalic discoloration, is the material from which, in several parts of the world, the richest specimens of porcelain ware are made. As it is, there are beds of it quite white, and pure in many places; one near Mr. Volney Dunnings on Rawson street, several on Whitehall street and in the excavations of the Central railroad.

The feldspar of Atlanta and surrounding country is, however, perfectly free from lime, and has only, here and there, a little magnesia. In a few localities magnesia appears as a carbonate, and when iron is, also, notably present, the springs and wells are both hard and calyebate, such as Ponce de Leon and the West End spring.

The temporary hardness of water is due to the presence of either the lime carbonate or that of magnesia, but this does not detract, a particle, from the wholesomeness of it. They only make it less valuable for manufactory and laundry purposes. It is claimed that the magnesia of Ponce de Leon, is a sulphate; if, so, the water of that famous spring is permanently hard, that is, not to be rendered soft by boiling, as the temporary hardness of the carbonate of lime or magnesia. This renders it the more valuable as a mineral spring with the tonic effects of its carbonate of iron.

Old citizens, such as the venerable Dr. Joseph Thompson, Judge Ezzard, Mr. Jonathan Norcross and others, under whose eyes, Atlanta has developed from a cross roads village, into a great metropolis, can testisy that there was no better drinking water in the world than that which flowed from the hills and valleys of her primitive site. Indeed it appeared to possess a virtue more subtile than that which imparts mere physical health, for according to a distinguished writer of the present century, this water is born of the very same geological formation, which in Virginia, the Carolinas, and the granite hills of New Hampshire nurtured the moral and intellectual greatness of the most remarkable men and women who have adorned American history.

Bog and rotten limestone water, was never known to develop a truly great man.

It has been before remarked that the chemical properties of a potable water are largely due to the nature of the soil through which it flows. This exceedingly porous, disintegrated feldspathic soil of Atlanta is everything that could be desired in relation to a wholesome supply of drinking water, so long as it retained the surroundings of a country community; but the subsequent development of the cross roads village into a great city has changed every condition. The loose, open soil gives ready passage, now into the springs and wells, to all the mineral or organic impurities with which the surface may be saturated; and this saturation is increasing and extending, every year, commensurate with the city's growth.

There is scarcely a single old well in the central, business part of the city that is not, now, so greatly contaminated from this source as to be too filthy and unwholesome for further use. The famous central springs with all their historical associations of lang syne, of which the old settlers sometimes love to talk, have long ago disappeared.

This brings up for consideration the wells which still supply in part the city with potable water, and the quality of water afforded by the public water system. Are the waters from these central wells palatable and wholesome? In order to test them fairly in this respect, it will be necessary to take the water undisguised by carbonic acid, delicate flavors, beer or other liquors. By careful examination, I find that from a few of them, palatable, and from nearly all, bright, sparkling waters may be got, but not one of them is absolutely safe, and a few are abominably filthy—reeking with organic matter.

This fact was made apparent not only by the microscope, to which I subjected them, but by chemical and physical analysis. I have now before me samples of water from the following wells in and out of the city: The well on Broad street between the bridge and Hunter street; a well at Jones' boarding house, 112, Decatur street; the well behind the arched entrance to the Markham House; the well in the City Hall Park; the famous well on Dr. Boring's lot, Forsyth street; the well on Mr. McNaught's place at the southern extremity of Washington street, and a well at the western end of Fair street. I took samples of these two last wells, to serve as a standard of purity to the others.

No exhaustive analysis has been attempted; this as is well known to every intelligent chemist, is very expensive, and was by no means essential to my purpose, which was simply to ascertain the comparative purity of our city's potable water, especially the presence or absence of noxious organisms, and decomposing organic matter in solution.

To be continued.

---

Dr. E. R. Palmer, Prof. Physiol. and Phys. Diag., Univ. of Louisville, says: "I regard *Bromidia* as a most excellent and reliable preparation."

